# Altered serum Zinc and Copper in Iranian Adults who were of normal weight but metabolically obese

**DOI:** 10.1038/s41598-019-51365-9

**Published:** 2019-10-16

**Authors:** Susan Darroudi, Narges Fereydouni, Maryam Tayefi, Habibollah Esmaily, Fatemeh Sadabadi, Zahra Khashyarmanesh, Batool Tayefi, Hamideh Moalemzadeh Haghighi, Ameneh Timar, Amir Hooshang Mohammadpour, Kayhan Gonoodi, Gordon A. Ferns, Seyed Javad Hoseini, Majid Ghayour-Mobarhan

**Affiliations:** 1 Student Research Committee, Faculty of Medicine, Mashhad University of Medical Sciences, Mashhad, Iran; 2 Norwegian Center for e-health Research, University hospital of North Norway, Tromsø, Norway; 3 Department of Biostatistics, School of Health, Mashhad University of Medical Sciences, Mashhad, Iran; 4 Department of Medicinal chemistry, School of pharmacology, Mashhad University of Medical Sciences, Mashhad, Iran; 5 Preventive Medicine and Public Health Research Center, Iran University of Medical Sciences, Tehran, Iran; 6 Faculty of Basic Science, Hakim Sabzevary University Sabzevar, Sabzevar, Iran; 7 Pharmaceutical Research Center, Pharmaceutical Institute Technology, Mashhad University of Medical Sciences, Mashhad, Iran; 8 Department of Clinical Pharmacy, School of Pharmacy, Mashhad University of Medical Sciences, Mashhad, Iran; 9 Department of Nutrition, Faculty of Medicine, Mashhad University of Medical Sciences, Mashhad, Iran; 10 Brighton & Sussex Medical School, Department of Medical Education, Falmer, Brighton, Sussex BN1 9PH UK; 11 Department of Biotechnology, School of Medicine, Mashhad University of Medical Sciences, Mashhad, Iran; 12 Metabolic Syndrome Research Center, School of Medicine, Mashhad University of Medical Sciences, Mashhad, Iran

**Keywords:** Dyslipidaemias, Epidemiology

## Abstract

Metabolically obese normal weight (MONW) individuals are potentially at increased risk of developing metabolic syndrome. Serum zinc and copper concentrations were assessed in individuals with MONW to determine whether MONW is associated with altered serum zinc and/or copper status. Normal weight subjects (total n = 2419; 1298 men and 1121 women), were recruited as part of Mashhad Stroke and Heart Association Disorder (MASHAD) Study cohort. They were divided into two groups according to the presence or absence of MetS, defined using IDF criteria. Serum zinc and copper concentrations were determined by atomic absorption. Of the 2419 normal weight adults, 377 had MetS. Of this group, 53.7% and 49.7% had a serum zinc <70 µg/dl (Q1) (p = 0.001) or a serum copper <79 µg/dl (Q1) respectively. Furthermore, 27.3% had a serum copper >131 µg/dl (Q4) (p = 0.034), and 18.8% had a serum zinc >95 µg/dl (Q4). Logistic regression analysis was performed to determine the odds ratio (OR) for an association of serum zinc, copper and zinc to copper ratio with MetS in normal weight subjects. The subjects with a serum zinc >95 µg/dl (Q4) had 0.386 [OR: 0.614(95%CI 0.457–0.823)] lower chance of MetS (p = 0.001) and the subjects with a serum copper >131 (Q4) had OR 1.423 (95% CI: 1.09–1.857) higher chance of MetS (p = 0.009). These data remained significant after adjustment for age and sex, for serum zinc and copper, respectively. Furthermore, our results strongly suggested that zinc and copper were the independent risk factor for metabolic syndrome in normal weight subjects. There is an imbalance between serum copper and zinc concentrations among individuals with MONW when compared with normal BMI individuals without MetS. This may increase the risk of individuals with MONW developing conditions associated with this imbalance, such as diabetes and cardiovascular disease.

## Introduction

Normal weight but metabolically obese individuals (MONW) are individuals whose BMI is within the normal, reference range, but who have metabolic disorders, such as impaired glucose tolerance, hypertension, or hypertriglyceridemia, which may be improved with caloric restriction^1^. Studies have shown that among individuals of normal weight, the risk of metabolic syndrome may be genetically determined^2^. MONW women have been shown to have a higher total body, abdominal and visceral fat, and lower levels of physical activity than women of normal weight, without metabolic obesity^3^.

It has been proposed that oxidative stress is a central mechanism for the complications in MetS, because there is a disturbance in the redox pathway to all risk factors of metabolic syndrome^4,5^. Copper (Cu) and zinc (Zn) are important trace elements, that act as structural ions in proteins, hormones, and receptors and as cofactors in numerous enzymatic reactions^6^. They are structurally important ions for superoxide dismutase (SOD)^7^ and may also reduce oxidative stress by induction of metallothionine synthesis^8,9^. Because of their pivotal role in the redox mechanisms, an imbalance may lead to an increased susceptibility to oxidative damage^10–12^.

Zn has a role in insulin signaling, synthesis, storage, and release^13^. Therefore, Zn deficiency may be associated with increased oxidative stress, abnormal insulin metabolism, central obesity, blood pressure, and triglyceride and HDL levels, which are important factors in the pathophysiology of diabetes and MetS^14,15^. It is proposed that a high dietary Zn intake may be protective against MetS^16,17^. Whilst the unbound form of Cu can participate in the generation of ROS and irreversibly bind to thiol group of proteins. This accumulation of Cu may potentiate ROS mediated damage in biological systems^18^.

It has been hypothesized that the balance between Cu and Zn has an impact in the antioxidant mechanisms and the development of MetS^15,19–21^. We aimed to evaluate the association between Cu and Zn status with the presence of MetS in Iranian population with a normal BMI to investigate whether Zn and Cu status in these subjects with a normal BMI may be associated with metabolic obesity.

## Methods

### Study population

According to the definition of normal weight but metabolically obese individuals (MONW), normal weight subjects (n = 2419; 1298 men and 1121 women) with a body mass index (BMI) of 18.5 to <25 kg/m^2^ were recruited as part of Mashhad Stroke and Heart Association Disorder (MASHAD) study. They were originally identified using a cluster randomized methodology during 2007–2008, as described previously^22^. Informed consent was obtained from all participants, and the study was been approved by the Ethics Committee of the Mashhad University of Medical Sciences (MUMS) (MASHAD study code: 85134)^22^. In addition, all procedures, protocols and experiments were performed in accordance with the MUMS guidelines for using humans in research project.

Data required for inclusion, included marital and occupational status, education level; exclusion criteria included medication use, comorbid conditions and inflammatory disease, as reported previously^22^

These subjects were then divided into groups with and without MetS, defined by the Harmonized definition of the Metabolic Syndrome and IDF criteria^23,24^.

### Demographic, anthropometric and metabolic data

For all subjects, height (in cm), weight (in kg), body mass index (in kg/m^2^) and waist circumference were measured. Height and weight were measured in centimeters and the nearest 0.1 cm with a stadiometer (SECA 217, Hamburg, Germany) and calibrated digital balance in kilogram scale (SECA 813, Hamburg, Germany) to the nearest 0.1 kg and, respectively. Waist and hip circumferences were measured to the nearest 0.1 cm.

Systolic and Diastolic Blood Pressure (SBP and DBP) were measured using a standard sphygmomanometer, twice in exactly the same manner. BP was measured using the left arm with individuals remaining seated after 15 minutes as described previously^25^. We took the third measurement and averaged the two closest readings, if the first two readings differed by >15 mmHg for DBP or >25 mmHg for SBP^22^.

### Biochemical analysis

Blood samples were collected from subjects between 8 and 10 a.m. by venipuncture of an antecubital vein after 14 h overnight fasting. The samples were collected in vacuum tubes (20 ml) from participants in a seated position, according to a standard protocol. Biochemical parameters, consisting of serum total cholesterol (TC), high-density lipoprotein cholesterol (HDL-C), low-density lipoprotein cholesterol (LDL-C) and triglycerides (TG), C-reactive protein (CRP) and fasting blood glucose (FBG) were determined as previously described^26^.

### Measurement of serum Zn and Cu concentrations

Serum samples were diluted with water at a ratio of 1:10. Flame atomic absorption (Varian AA240FS) was used to measure serum Zn and Cu concentrations, according to Taylor *et al*., and Ghayour *et al*., as previously reported^27,28^. Zn and Cu standard curves were constructed using a Zn and Cu standard (Merck and Co. Pharmaceutical Company). The accuracy of methods was 93 ± 4.8% for Zn and 95 ± 3.75% for Cu, estimated by measuring a certified reference material (Merck KGaA 64271 Darmstadt, Germany) containing a known amount (1000 ± 2 mg/L) of Zn and Cu. The intra-assay and inter-assay coefficient of variation (CV) were 1.5 ± 0.2% and 2.6 ± 0.4% for Zn and 1.3 ± 0.12% and 2.11 ± 0.32% for Cu. The limit of detection was less than 0.1 mg/L.

### Statistical analysis

SPSS version 18(SPSS Inc. Chicago, IL, USA) was used for all statistical analyses. The normality of the data was assessed using the Kolmogorov-Smirnov test. Descriptive statistics including mean, frequency, and standard deviation (SD) were defined for all variables and expressed as mean ± standard deviation (SD) for variables with normally distribution or median ± IQR for not normally distributed variables. The differences in the mean values of clinical and baseline demographics characteristics between two groups and metabolic syndrome components were evaluated using analysis of covariance (ANCOVA) with age and gender as model covariates. For categorical parameters, a chi-square, or Fisher exact tests were used. Logistic regression analysis was used to evaluate the association of Zn, Cu and Zn to Cu ratio quartiles with metabolic syndrome in normal BMI subjects. All the analyses were two-sided and p- value < 0.05 was considered as significant. GraphPad Prism 6 for figures was used.

## Result

### Prevalence of metabolic syndrome in normal BMI subjects (MONW)

Of the 2419 normal weight adults in the group, 2042 did not have MetS and 377 were found to have MetS; the mean ages of the groups were 47.24 ± 7.94 and 49.82 ± 7.81 years, respectively. The prevalence of MetS among MONW individuals was 8.7% and 23.6% in men and women, respectively (Table 1) and there were significant differences in age and gender between the two groups.

**Table 1:** Anthropometric and biochemical characteristics of subjects with a normal BMI, according to the presence or absence of Metabolic Syndrome (n: 2419).

		Normal (n:2042)	MONW (n:377)	p-value
Prevalence % (population frequency)	2419(100)	2402(84.4%)	377 (15.6%)	
Age, y		47.24 ± 7.94	49.82 ± 7.81	<0.001
Sex, %	Male(1298),	1186(58.08%)	113(29.97%)	<0.001
	Female(1121),	856(41.92%)	264(70.03%)	
Smoking, %	Never (1586)	1303(63.81%)	265(70.29%)	0.024
	Former (252)	225 (11.02%)	27(7.16%)	
	Current (599)	514(25.17%)	85(22.55%)	
SBP, mm Hg		115.39 ± 18.45	125.46 ± 19.55	<0.001
DBP, mm Hg		75.86 ± 11.6	81.04 ± 12.14	<0.001
HDL-C, mg/dl		44.56 ± 10.23	39.74 ± 9.88	<0.001
Triglycerides, mg/dl		129(89–190)	138(94–190)	<0.001
Glucose, mg/dl		86.92 ± 43.57	100.99 ± 37.22	<0.001
waist circumference (cm)		84.21 ± 11.74	90.8 ± 11.98	<0.001
hs-CRP (mg/l)		1.2(0.75–2.12)	1.3(0.96–2.43)	0.374
Serum zinc (µg/dl)		86.78 ± 20.74	83.39 ± 18.1	0.019
Serum copper (µg/dl)		104.13 ± 36.27	108.26 ± 34.03	0.034
Zinc Copper Ratio		1.01 ± 0.72	0.926 ± 0.67	0.059

Data are presented as mean (SD) or interquartile range. Differences in variables among metabolic syndrome negative and positive subjects determined using ANCOVA analyses with age and gender included as model covariates; MONW: metabolic obese normal weight.

### General characteristics of the subjects

Anthropometric and biochemical characteristics of the subjects are shown in Table 1. There were 14.2% of current smokers, 10.7% of ex-smokers and 16.9% of non-smokers in the group with MetS and there were significant differences among these categories between the groups with and without MetS. The mean SBP and DBP, total cholesterol, triglyceride, LDL, glucose, waist circumference and serum Cu were significantly higher in the group with MetS (p < 0.05). Serum HDL, serum Zn and the Zn to Cu ratio were lower in the group with MetS (p < 0.05).

### Prevalence of metabolic syndrome according to Zn and Cu status

Of the individuals in the MetS group, 53.32% and 49.34% had serum Zn <70 µg/dl (Q1) or a serum Cu <79 µg/dl (Q1), respectively (Table 2). Of this group, 27.58% had a serum Cu >131 µg/dl (Q4), and 18.83% of subjects had serum Zn >95 µg/dl (Q4) (Table 2). There were differences between subjects with MetS and serum zinc and copper quartiles (p < 0.05).

**Table 2:** Prevalence of Metabolic Syndrome according to serum zinc and copper quartiles.

	Normal (n:2042)	MONW (n:377)	p-value
**Zinc**			
Quartile1 (<70 µg/dl)	982 (48.09%)	201 (53.32%)	0.001
Quartile2,3 (70–95 µg/dl)	487 (23.85%)	105 (27.85%)	
Quartile4 (>95 µg/dl)	573 (28.06%)	71 (18.83%)	
**Copper**			
Quartile1 (<79 µg/dl)	1122 (54.95%)	186 (49.34%)	0.034
Quartile2,3 (79–131 µg/dl)	475 (23.26%)	87 (23.08%)	
Quartile4 (>131 µg/dl)	445 (21.79%)	104 (27.58%)	

**Figure 1:**
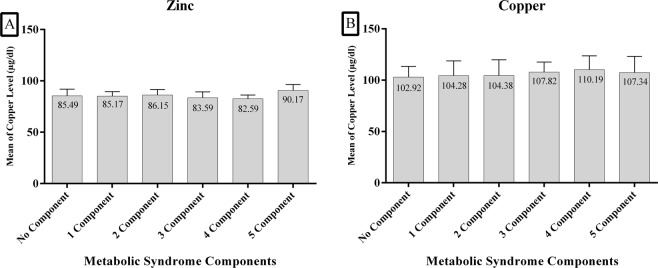
Serum zinc and copper according to Metabolic Syndrome components in normal BMI subjects.

**Figure 2:**
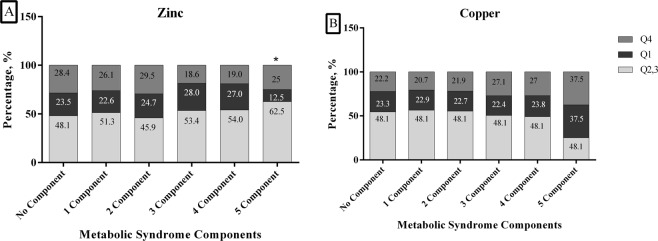
Serum Zinc (**A**) and Copper (**B**) quartiles according to Metabolic Syndrome components in normal BMI subjects. (Q1, Q2-3 and Q4 for zinc defined as Q1: zinc <70 µg/dl, Q2-3: zinc 70–95 µg/dl and Q4: zinc >95 µg/dl; Q1, Q2-3 and Q4 for copper defined as Q1: copper <79 µg/dl, Q2-3: copper 79–131 µg/dl and Q4: copper >131 µg/dl).

According to the number of components of MetS (0–5 components) that were present, mean serum Zn concentrations were 85.49, 85.17, 86.15, 83.59, 82.59 and 90.72 µg/dl, respectively (Fig. 1). There were no significant differences between mean serum of Zn and the increasing number of components of MetS. Figure 2 shows the percentage of individuals, (25%, 12.5% and 62.5%) with five components that were in the various quintiles (Q4, Q1 and Q2–3) of serum Zn, respectively. The corresponding values for those with 4 components were 19%, 27% and 54% in Q4, Q1 and Q2-3 of serum Zn, respectively. Finally, 18.6%, 28% and 53.4% of subjects had three components of MetS in Q4, Q1 and Q2-3 of serum Zn, respectively. There were significant differences between quartiles of serum Zn and the presence of number of components of MetS (p < 0.05) (Fig. 2).

The mean serum Cu values were 102.92, 104.38, 104.38, 107.82, 110.19 and 107.34 µg/dl, in the groups with increasing numbers of components of MetS (0–5), respectively (Fig. 1). No significant differences were observed between mean serum Cu in individuals and different numbers of components of MetS. Figure 2 shows that 27.1%, 22.4% and 50.5% of the subjects had three components that were in Q4, Q1 and Q2-3 of serum Cu, respectively. 27%, 23.8% and 49.2% of subjects had four components that were in Q4, Q1 and Q2-3 of Cu, respectively; 37.5%, 37.5% and 25% of subjects had five components categorized in Q4, Q1 and Q2-3 of Cu, respectively. There was no significant difference between quartiles of Cu and the number of components of MetS (p > 0.05) (Fig. 2B).

### Zn, Cu and Zn to Cu ratio indices as risk factors for MetS in normal weight subjects

Logistic regression analysis was performed to determine the odds ratio (OR) of the association between serum Zn, Cu, and Zn to Cu ratio with the presence of MetS in normal weight individuals from MASHAD cohort (Table 3). Univariate analysis for Zn, Cu and Zn to Cu ratio quartiles is shown in Table 3. Subjects with a serum Zn >95 µg/dl (Q4) had a 0.614 OR of MetS (CI 0.457–0.823, p < 0.05). In individuals with a serum Cu >131 µg/dl the OR of MetS was 1.423 (CI 1.09–1.857). These data were recalculated after adjustment for age and sex, OR 0.624 (CI 0.462–0.843) and 1.439(CI 1.092–1.896) in Q4 of serum Zn and Cu, respectively (Table 3).

**Table 3:** Unadjusted and Multivariate-Adjusted of Odds Ratios of the MONW according to serum zinc and copper quartiles in normal BMI subjects.

	Unadjusted	p-value	Adjusted by sex and age	p-value
**Zinc (µg/dl)**				
Quartile 2,3 (72–95)	Reference		Reference	
Quartile 1 (<72)	1.061(0.814–1.382)	0.662	1.049(0.798–1.379)	0.73
Quartile 4 (>95)	0.614(0.457–0.823)	0.001	0.624(0.462–0.843)	0.002
**Copper**				
Quartile 2,3 (79–131)	Reference			
Quartile 1 (<79)	1.123(0.85–1.483)	0.414	1.114(0.836–1.484)	0.46
Quartile 4 (>131)	1.423(1.09–1.857)	0.009	1.439(1.092–1.896)	0.01
**Zinc Copper Ratio**				
Quartile 2,3 (0.61–1.09)	Reference		Reference	
Quartile 1 (<0.61)	1.354(1.04–1.758)	0.024	1.312(1.011–1.72)	0.049
Quartile 4 (>1.09)	0.914(0.687–1.216)	0.53	0.909(0.677–1.219)	0.52

## Discussion

We have investigated the relationship between serum Cu and Zn in subjects with MONW who have a normal BMI (<25Kg/m^2^) but have the criteria for MetS as defined by the IDF criteria. The prevalence of MONW was found to be approximately 16% overall in an Iranian population, with a significant difference between gender (women, 23.6% vs. men, 8.7%, p < 0.001). The prevalence of MetS in male and female individuals in the entire MASHAD cohort study (n = 9761) was 27.8% and 40.8%, respectively; in the subgroup with NOMW these values were lower, but remained higher in women. Janghorbani *et al*. have reported excess body weight appears to be common in Iran. Women had overweight and abdominal obesity more frequently than men^29^. Esmaillzadeh *et al*. showed that there was significant associations among major dietary patterns, general obesity, and central adiposity in a Middle-Eastern country^30^. Bahrami *et al*. showed that the prevalence of obesity, overweight, and hypertension in Iran is as high as the US. However, Iranian women are more obese than American women and Iranian men are less obese than their American counterparts. Iranian women have higher mean WHR than what WHO has defined in 19 other populations^31^. Sarrafzadegan *et al*. showed that metabolic syndrome is highly prevalent in the Iranian population, notably in women living in urban areas. Abdominal obesity and dyslipidemia characterize this syndrome. Implementing community-based strategies for lifestyle change is of great significance^32^.

Park *et al*. have reported that 4.6% of normal weight men and 6.2% normal weight women (with a BMI <27) had MetS according to APTIII criteria in a population from the USA in 2003^33^. St-Onge *et al*. showed that the prevalence of MONW, depending on sex and ethnicity, differs from 10–18% in men and 15–23% in women (BMI 25–27)^2^. Meigs *et al*. showed that prevalence of MONW differs from 7.1–7.7% on the basis of MetS criteria definition (ATPIII or HOMA)^34^. Taking these results together, suggest that the prevalence of MONW may differ depending on ethnicity, sex, age, and the criteria used for MetS. The data also suggests that recommendations for weight loss regardless of the metabolic syndrome components are not sufficient. This is in accordance with a report noting that the incidence of diabetes, hypertension, and coronary heart disease increases well below the normal BMI cutoff of 25.0 kg/m^2,35^.

Because of the importance of Zn and Cu in human metabolism, our study has examined serum Zn and Cu concentrations in individuals with MONW. Although obesity is one of the most important features of metabolic syndrome, in this study we have examined serum zinc and copper concentrations in a subgroup of individuals with MetS with MNOW, because although they appear to be healthy, they are likely to be susceptible to cardiovascular disease and diabetes. We found that serum Cu and Zn were associated with the prevalence of MetS in normal BMI subjects in an Iranian population. Several studies have reported a relationship between serum Zn and Cu levels and MetS^15,19–21^, however these relationships have been inconsistent in various populations. There are few studies on the association of Zn and Cu levels with metabolically obese normal weight individuals (MONW). In the fifth Korea National Health and Nutrition Examination Survey (KNHANES V) cohort of 1813 subjects, it was shown that lower quartile levels of serum Zn show a higher level of fasting blood glucose (FBG) and insulin resistance compared to higher quartile levels. MONW individuals have lower levels of Zn compared to normal and healthy metabolic subjects (MHNW), while serum Zn level did not differ significantly from the level in obese subjects with a normal metabolic profile^36^. Zinc plays an important role in normal functioning of the pancreatic beta cells. Insulin is stored in vesicles or secretary granules, where two zinc ions are attached to six insulin monomers to form insulin hexameric structures^37^. Insulin production and effective packaging of vesicles depend on transfer of zinc to beta cells via ZnT8 transporter, a product of SLC30A8 gene. Changes in the activity of this transporter are associated with impaired response to insulin and contribute to the progression from glucose intolerance to type 2 diabetes^38,39^. Zinc ions and its multiple complexes have similar insulin functions. Zinc complexes activate insulin-signaling cascades through Akt/PKB, which leads to an increase in cellular GLUT4 and an increase in cellular glucose uptake. In animal models of type 2 diabetes, these complexes have a significant potential to reduce blood glucose, insulin serum, HbA1c, triglyceride, and total cholesterol, while improving glucose tolerance^40^. The present study also showed a significant difference in mean serum Zn (p = 0.019) between the MONW and normal subjects. The prevalence of MONW in quartile 1 (Q1) of Zn (<70 µg/dl) was associated with the highest prevalence of MONW (Table 3).

There are no previous studies that assess serum copper in MNOW, but there are studies that show higher levels of copper in people with MetS^41^ and cardiovascular disease^42^. The present study also showed a significant difference in mean serum Cu (p = 0.034) between the MONW and normal subjects. The prevalence of MONW in quartile 4 (Q4) of serum Cu (>131 µg/dl) was associated with a high prevalence of MONW than Q1 and Q2, 3 (Table 3).

Alterations in Cu and Zn status may be important in determining oxidative stress^43,44^. On the other hand, Cu and Zn may be important for antioxidant processes; they are essential for the biosynthesis of the antioxidant enzyme Cu-Zn superoxide dismutase (SOD), but may also participate in pro-oxidant reactions and lipid oxidation. Another reason may be due to the relationship between Zn and insulin resistance. Zn is introduced by Zn transporter 8 into pancreatic beta cells and plays an important role in the synthesis, storage, secretion and function of insulin^45^. Several mechanisms have been proposed to explain Cu-induced cytotoxicity^46,47^. The basis of most of these is the tendency of free Cu ions to participate in the formation of reactive oxygen species (ROS). In the presence of superoxide (O_2_°^−^) or reducing agents such as ascorbic acid or GSH, both cuprous (Cu^1+^) and cupric (Cu^2+^) ions can be reduced to Cu which is capable of catalyzing hydrogen peroxide (H_2_O_2_) to hydroxyl radicals (OH°)^46,47^. The hydroxyl radicals are the most powerful oxidizing radicals likely to be created in biological systems, and is able to react with every biological molecule^48^. Cu is also a powerful catalyst of LDL oxidation and may create atherogenic form of LDL^49^. It is likely that even in the absence of chelating agents, Cu^2+^ linkage to histidine residue a polipoprotein B-100 of LDL molecule may act as a reducing agent to form Cu^50^. In addition to free Cu ions, ceruloplasmin serve as a source of free Cu which contains seven atoms of Cu in each molecule^51^. In conditions of oxidative stress, nitric oxide and superoxide may destroy the transport protein ceruloplasmin and release Cu ions to be involved in LDL oxidation^52^. It is also shown that high density lipoprotein (HDL) may be more sensitive to Cu-induced oxidation than LDL at low Cu concentration because of increased tocopherol-mediated peroxidation^53^.

We found that the Zn to Cu ratio was not significantly associated with MONW in Iranian adults. In studies on the relationship between Zn, Cu levels and MetS, it has been shown that the level of Zn to Cu ratio may provide a more accurate prediction than levels of serum Zn or Cu alone. Xu *et al*. explore that looking in either Zn or Cu may result in mistake since the evaluation of each element individually depends on their dietary intake difference^54^.

We have found that there is an imbalance in the levels of serum Cu and Zn among individuals with MONW in comparison with normal BMI. It is unclear whether these changes may play a role in the pathogenesis of MetS in normal BMI individuals, or they are a consequence of other metabolic abnormalities, including insulin resistance. This requires an investigation of the relationship between all components of the metabolic syndrome and the level of Zn and Cu. On the other hand, as the amount of these trace elements changes in the serum of MNOW individuals, this can be considered as a biomarker in identifying of susceptible people to metabolic syndrome.
